# Supplementary material for: Reduced mu opioid receptor availability in schizophrenia revealed with [11C]-carfentanil positron emission tomographic Imaging
Source: Nat Commun. 2019 Oct 3;10:4493. doi: 10.1038/s41467-019-12366-4 (PMC6776653; doi:10.1038/s41467-019-12366-4)
Supplement: Supplementary file 1 — Supplementary Information [file 41467_2019_12366_MOESM1_ESM.docx]

**Supplementary material: Reduced mu opioid receptor availability in schizophrenia revealed with [11C]-carfentanil Positron Emission Tomographic Imaging, Ashok et al.**

**Supplementary Figure 1: There was no significant association between striatal MOR availability with negative symptom severity in schizophrenia patients (PANSS-negative symptom subscale (r: 0.07, p=0.78).**

**Supplementary Figure 2: Standardised uptake values (SUV) in the occipital cortex grey matter (GM; the reference region)) in patients and controls (red line represents patients and blue line represents healthy controls), showing no significant group difference (p>0.05).**

**
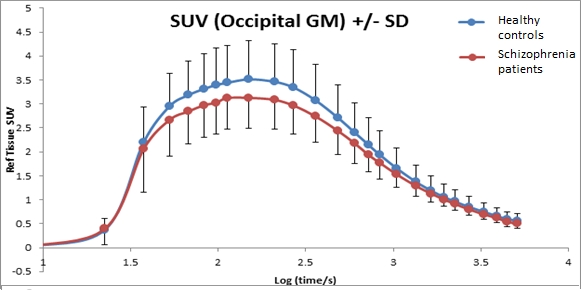
**

**Supplementary Figure 3:** There was no association between striatal MOR availability and antipsychotic dose expressed as the chlorpromazine equivalent dose (r=0.06, p=0.82).


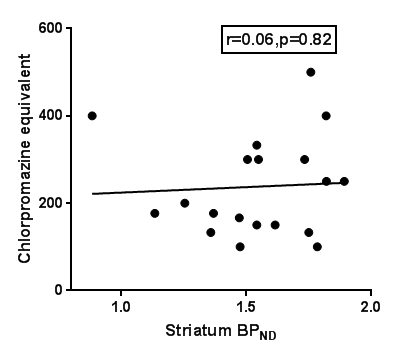


| Participant ID | **Medication (daily dose unless otherwise stated)** |
| --- | --- |
| 101 | Olanzapine 12.5 mg |
| 102 | Olanzapine 25 mg |
| 104 | Risperidone depot 50 mg every 2 weeks |
| 105 | Olanzapine 20 mg |
| 106 | Zuclopenthixol 500mg every 2 weeks |
| 107 | Amisulpride 400mg |
| 108 | Aripiprazole 25mg |
| 109 | Olanzapine 20 mg |
| 110 | Risperidone 3mg |
| 111 | Olanzapine 15 mg |
| 112 | Paliperidone 75mg once a month |
| 113 | Aripiprazole depot 400mg monthly |
| 114 | Risperidal Consta 25mg every two weeks |
| 115 | Aripiprazole 10mg |
| 116 | Flupenthixol 60 mg two weekly |
| 117 | Flupenthixol 40mg once a month |
| 118 | Clozapine 150mg |
| 119 | Aripirprazole depot 400mg monthly |
| 120 | Olanzapine 15mg |

**Supplementary Table 1: Medication details of patients**

**Supplementary Table 2: Affinity of antipsychotics to µ-Opioid receptor**

| ***Drug*** | ***Ki*** | ***Species*** | ***Brain region*** | ***Hot ligand*** | ***Reference*** |
| --- | --- | --- | --- | --- | --- |
| **Clozapine** | 1000 | Rat | Forebrain | 3H-Sufentanil | ^1^ |
| **Haloperidol** | 1000 | Rat | forebrain | 3H-Sufentanil | ^1^ |
| **Olanzapine** | 1000 | Rat | forebrain | 3H-Sufentanil | ^1^ |
| **Quetiapine** | 1000 | Rat | forebrain | 3H-Sufentanil | ^1^ |
| **Risperidone** | 1000 | Rat | forebrain | 3H-Sufentanil | ^1^ |
| **Sertindole** | 1000 | Rat | forebrain | 3H-Sufentanil | ^1^ |
| **Ziprasidone** | 1000 | Rat | forebrain | 3H-Sufentanil | ^1^ |
| **Zotepine** | 1000 | Rat | forebrain | 3H-Sufentanil | ^1^ |
| **Aripiprazole** | >10,000 | Human | cloned | 3H-Diprenorphine | ^2^ |
| **Amisulpride** | >10,000 | Human | cloned | 3H-DAMGO | ^3^ |
| **Iloperidone** | >10,000 | Human | cloned | 3H-NALOXONE | ^4^ |

**Supplementary Table 3: Volume differences between patients and controls in hedonic regions (in mm^3^).**

| **Regions** | **Patients**  **Mean ± SD** | **Controls**  **Mean ± SD** | **t** | **df** | **Sig. (2-tailed)** |
| --- | --- | --- | --- | --- | --- |
| **Anterior cingulate cortex** | 31228 ± 2108 | 34003± 5115 | -2.193 | 37 | .035 |
| **Amygdala** | 3611 ± 306 | 3884 ± 384 | -2.446 | 37 | .019 |
| **Orbitofrontal cortex** | 26978 ±3399 | 30174 ±4384 | -2.535 | 37 | .016 |
| **Insula** | 12962 ±1078 | 13959 ± 1671 | -2.199 | 37 | .034 |

SD: standard deviation

**Reference:**

1. Schotte A, Janssen PF, Gommeren W, et al. Risperidone compared with new and reference antipsychotic drugs: in vitro and in vivo receptor binding. *Psychopharmacology.* 1996;124(1-2):57-73.

2. Shapiro DA, Renock S, Arrington E, et al. Aripiprazole, a novel atypical antipsychotic drug with a unique and robust pharmacology. *Neuropsychopharmacology : official publication of the American College of Neuropsychopharmacology.* 2003;28(8):1400-1411.

3. Abbas AI, Hedlund PB, Huang XP, Tran TB, Meltzer HY, Roth BL. Amisulpride is a potent 5-HT7 antagonist: relevance for antidepressant actions in vivo. *Psychopharmacology.* 2009;205(1):119-128.

4. Kalkman HO, Subramanian N, Hoyer D. Extended radioligand binding profile of iloperidone: a broad spectrum dopamine/serotonin/norepinephrine receptor antagonist for the management of psychotic disorders. *Neuropsychopharmacology : official publication of the American College of Neuropsychopharmacology.* 2001;25(6):904-914.
