# Supplementary material for: Reduced mu opioid receptor availability in schizophrenia revealed with [11C]-carfentanil positron emission tomographic Imaging
Source: Nat Commun. 2019 Oct 3;10:4493. doi: 10.1038/s41467-019-12366-4 (PMC6776653; doi:10.1038/s41467-019-12366-4)
Supplement: Supplementary file 3 — Reporting Summary [file 41467_2019_12366_MOESM3_ESM.pdf]

nature research

Corresponding author(s): Abhishekh Ashok

Last updated by author(s): 17

Reporting Summary

Nature Research wishes to improve the reproducibility of the work that we publish. This form provides structure for consistency and transparency in reporting. For further information on Nature Research policies, see Authors & Referees and the Editorial Policy Checklist.

Statistics

For all statistical analyses, confirm that the following items are present in the figure legend, table legend, main text, or Methods section.

n/a	Confirmed	
☐	☒	The exact sample size (*n*) for each experimental group/condition, given as a discrete number and unit of measurement
☐	☒	A statement on whether measurements were taken from distinct samples or whether the same sample was measured repeatedly
☐	☒	The statistical test(s) used AND whether they are one- or two-sided<br>*Only common tests should be described solely by name; describe more complex techniques in the Methods section.*
☐	☒	A description of all covariates tested
☐	☒	A description of any assumptions or corrections, such as tests of normality and adjustment for multiple comparisons
☐	☒	A full description of the statistical parameters including central tendency (e.g. means) or other basic estimates (e.g. regression coefficient) AND variation (e.g. standard deviation) or associated estimates of uncertainty (e.g. confidence intervals)
☐	☒	For null hypothesis testing, the test statistic (e.g. *F*, *t*, *r*) with confidence intervals, effect sizes, degrees of freedom and *P* value noted<br>*Give P values as exact values whenever suitable.*
☒	☐	For Bayesian analysis, information on the choice of priors and Markov chain Monte Carlo settings
☒	☐	For hierarchical and complex designs, identification of the appropriate level for tests and full reporting of outcomes
☐	☒	Estimates of effect sizes (e.g. Cohen's *d*, Pearson's *r*), indicating how they were calculated

Our web collection on statistics for biologists contains articles on many of the points above.

Software and code

Policy information about availability of computer code

Data collection	No software has been used.
Data analysis	MIAKAT software description has been given.

For manuscripts utilizing custom algorithms or software that are central to the research but not yet described in published literature, software must be made available to editors/reviewers. We strongly encourage code deposition in a community repository (e.g. GitHub). See the Nature Research guidelines for submitting code & software for further information.

Data

Policy information about availability of data

All manuscripts must include a data availability statement. This statement should provide the following information, where applicable:

- Accession codes, unique identifiers, or web links for publicly available datasets
- A list of figures that have associated raw data
- A description of any restrictions on data availability

Data available on request

Field-specific reporting

Please select the one below that is the best fit for your research. If you are not sure, read the appropriate sections before making your selection.

☒ Life sciences ☐ Behavioural & social sciences ☐ Ecological, evolutionary & environmental sciences

For a reference copy of the document with all sections, see nature.com/documents/nr-reporting-summary-flat.pdf

October 2018

Life sciences study design

All studies must disclose on these points even when the disclosure is negative.

Sample size	For the [^{11}C] Carfentanil group using two tailed t-test, anticipating an effect size of d=1.05 with alpha = 0.05, power = 0.8, we would require n=16 participants in each group using G* software (Kennedy et al., 2006) .
Data exclusions	One scan was excluded due to structural abnormality.
Replication	N/A
Randomization	N/A
Blinding	N/A

Reporting for specific materials, systems and methods

We require information from authors about some types of materials, experimental systems and methods used in many studies. Here, indicate whether each material, system or method listed is relevant to your study. If you are not sure if a list item applies to your research, read the appropriate section before selecting a response.

Materials & experimental systems

n/a	Involved in the study	
☒	☐	Antibodies
☒	☐	Eukaryotic cell lines
☒	☐	Palaeontology
☒	☐	Animals and other organisms
☒	☐	Human research participants
☒	☐	Clinical data

Methods

n/a	Involved in the study	
☒	☐	ChIP-seq
☒	☐	Flow cytometry
☐	☒	MRI-based neuroimaging

Magnetic resonance imaging

Experimental design

Design type	Structural imaging
Design specifications	N/A
Behavioral performance measures	N/A

Acquisition

Imaging type(s)	structural analysis for PET coregistration
Field strength	3T
Sequence & imaging parameters	High resolution T1 weighted volumes were acquired using a 3T MR scanner (Magneton Trio Syngo MR B13 Siemens 3T; Siemens AG, Germany) and a magnetization prepared rapid gradient echo (MPRAGE) sequence (TR = 2300 ms, TE = 2.98 ms, TI = 900 ms, flip angle = 9°, field of view = 256 mm, image matrix = 240 x 256) with a resolution of 1 mm isotropic. For the volume, 160 abutting straight sagittal slices were collected in an interleaved right to left manner, resulting in whole head coverage. Parallel imaging using Generalized Auto calibrating Partially Parallel Acquisition (GRAPPA) with an acceleration factor of 2 was performed.
Area of acquisition	Whole

Diffusion MRI ☐ Used ☒ Not used

Preprocessing

Preprocessing software	SPMM
Normalization	*If data were normalized/standardized, describe the approach(es): specify linear or non-linear and define image types used for transformation OR indicate that data were not normalized and explain rationale for lack of normalization.*

Normalization template | *Describe the template used for normalization/transformation, specifying subject space or group standardized space (e.g. original Talairach, MNI305, ICBM152) OR indicate that the data were not normalized.*

Noise and artifact removal | *Describe your procedure(s) for artifact and structured noise removal, specifying motion parameters, tissue signals and physiological signals (heart rate, respiration).*

Volume censoring | *Define your software and/or method and criteria for volume censoring, and state the extent of such censoring.*

Statistical modeling & inference

Model type and settings | *Specify type (mass univariate, multivariate, RSA, predictive, etc.) and describe essential details of the model at the first and second levels (e.g. fixed, random or mixed effects; drift or auto-correlation).*

Effect(s) tested | *Define precise effect in terms of the task or stimulus conditions instead of psychological concepts and indicate whether ANOVA or factorial designs were used.*

Specify type of analysis: ☒ Whole brain ☐ ROI-based ☐ Both

Statistic type for inference
(See Eklund et al. 2016) | *Specify voxel-wise or cluster-wise and report all relevant parameters for cluster-wise methods.*

Correction | *Describe the type of correction and how it is obtained for multiple comparisons (e.g. FWE, FDR, permutation or Monte Carlo).*

Models & analysis

n/a	Involved in the study
☒	☐ Functional and/or effective connectivity
☐	☒ Graph analysis
☒	☐ Multivariate modeling or predictive analysis

Graph analysis

MOR covariance analysis
In addition to regional alterations, it remains unknown if global brain MOR organisation is altered in schizophrenia. To assess this, we performed a correlation analysis for the whole brain and compared this for the two groups.117 Within each group, the correlation coefficients between [11C]-carfentanil BPND at each ROI with all other ROIs (125 ROIs defined in the Clinical Imaging Centre atlas105) were calculated and z values derived using the Fisher z-transformation to derive a global correlation matrix. This matrix was considered as a covariance network, where nodes are the ROIs and interregional correlations are the edges. The matrix consisted of 7750 edges. To determine the difference in the strength of connectivity of the edges between patient and control groups, each edge within these networks was compared between groups using permutation testing in MATLAB (100,000 permutations of group labels). To correct for the large number of partially-dependent comparisons, the network based statistic was used, with a primary threshold of alpha=0.05.118 To identify primary contributors to the effect between groups, we conducted a further analysis using a more conservative threshold, alpha=0.001, consistent with the approach used in previous imaging studies using network based stastistics117-119.
